# A homozygous splice-site variant in *ZMYND10* causes primary ciliary dyskinesia with primary infertility in a Chinese family

**DOI:** 10.3389/fmed.2026.1838497

**Published:** 2026-05-08

**Authors:** Jie He, Xiangyang Lu, Manqing Guo, Binyi Yang, Xianglin Zhou, Ying Liu, Hui Fan, Danhui Yang, Hong Luo

**Affiliations:** 1 Department of Pulmonary and Critical Care Medicine, The Second Xiangya Hospital, Central South University, Changsha, Hunan, China; 2 Research Unit of Respiratory Disease, Central South University, Changsha, Hunan, China; 3 Clinical Medical Research Center for Pulmonary and Critical Care Medicine in Hunan Province, Changsha, Hunan, China; 4 Diagnosis and Treatment Center of Respiratory Disease in Hunan Province, Changsha, Hunan, China

**Keywords:** ciliary assembly, primary ciliary dyskinesia, primary infertility, splice-site variant, ZMYND10

## Abstract

Primary ciliary dyskinesia (PCD) is a monogenic disorder of motile cilia characterized by impaired mucociliary clearance and multisystem involvement. We describe a patient with bronchiectasis, chronic rhinosinusitis, situs inversus, and primary infertility with hoard the *ZMYND10* splice-site variant (NM_015896.4:c.511–1G>A). High-speed video microscopy (HSVM) demonstrated near-complete immotility of respiratory cilia. RT-PCR and cDNA sequencing revealed aberrant splicing with insertion of a 100-bp fragment, leading to a frameshift and a pre-mature termination codon predicted to trigger non-sense-mediated mRNA decay and truncate the C-terminal MYND domain. Consistently, *ZMYND10* transcript and protein levels were markedly reduced. Transmission electron microscopy demonstrated loss of both outer and inner dynein arms. Concordantly, immunofluorescence showed absence of DNAH5, supporting an outer dynein arm defect. In addition, single-headed inner dynein arm components were selectively disrupted, as evidenced by loss of DNALI1, whereas the double-headed IDAf component DNAH2 was preserved. Notably, GAS8 (DRC4) was absent despite preserved DRC1 expression, suggesting that GAS8 loss in this case was not simply secondary to DRC1 deficiency and may reflect broader perturbation of nexin–dynein regulatory complex organization. Collectively, these findings expand the phenotypic spectrum and molecular characterization of ZMYND10-related PCD and provide further insight into dynein arm assembly and axonemal organization.

## Introduction

Primary ciliary dyskinesia (PCD) is a rare monogenic disorder, most commonly inherited in an autosomal recessive manner, and more than 50 disease-causing genes have been identified to date (1, 2). Clinically, adult patients with PCD frequently present with bronchiectasis and chronic rhinosinusitis due to recurrent respiratory tract infections, and may also exhibit extrapulmonary manifestations such as situs inversus and infertility (1). Current research indicates that major pathogenic mechanisms of PCD involve primary defects in the function and/or structure of motile cilia, or reduced generation of multiple motile cilia (3, 4). Normal mucociliary clearance in the airways relies on the coordinated, directional beating of cilia (5). When pathogenic variants lead to abnormalities in the classical “9 + 2” axonemal ultrastructure, ciliary motility becomes impaired, ultimately resulting in disease onset and progression.

Currently, defects in outer dynein arm (ODA) function represent common pathogenic mechanism of PCD, frequently implicated genes including *DNAH5* and *DNAH11* (2). In addition, a subset of genes is responsible for the assembly of both outer and inner dynein arms (ODAs and IDAs). When these genes are functionally impaired, proper dynein arm assembly is disrupted, ultimately leading to structural loss of dynein arms (6). ZMYND10, a member of the zinc finger MYND domain-containing protein family, plays a key role in this process. Patients with loss-of-function mutations in *ZMYND10* exhibit typical clinical features of PCD, and ultrastructural analysis of respiratory cilia demonstrates combined absence of both ODAs and IDAs (7, 8).

In this study, we provide an expanded clinical and molecular characterization of a patient harboring the *ZMYND10* splice-site variant (NM_015896.4:c.511–1G>A), which was previously described by our group in preliminary abstract form (9). Specifically, we further demonstrate the RNA-level splicing consequence of this variant by RT-PCR and cDNA sequencing, confirm loss of *ZMYND10* expression at the protein level, and extend the axonemal phenotyping by showing preserved DRC1, RSPH3, and SPEF2 despite loss of GAS8. Female primary infertility has been only sporadically noted in *ZMYND10*-related PCD and remains underrecognized (8, 10). Together, these findings expand the phenotypic and molecular spectrum of *ZMYND10*-related PCD and provide additional clues to the molecular consequences of ZMYND10 dysfunction.

## Methods

### Human subjects

The proband and available family members were recruited from the Second Xiangya Hospital of Central South University (Changsha, China) as part of a clinical genetic investigation. Detailed phenotypic evaluations included chest and paranasal sinus computed tomography (CT), sputum culture, echocardiography, nasal nitric oxide measurement, and chromosomal karyotype analysis, as clinically indicated. The study was approved by the Ethics Committee of the Second Xiangya Hospital and conducted in accordance with institutional and international ethical standards. Written informed consent was obtained from the patient prior to participation.

### Whole-exome sequencing (WES) and bioinformatic analysis

Peripheral blood samples were collected from the proband and available family members after obtaining written informed consent. Genomic DNA was extracted using the QIAamp DNA Blood Mini Kit (Qiagen, Valencia, CA) following the manufacturer's protocol. Briefly, exonic regions of the patient's genomic DNA were enriched using the Agilent SureSelect Human All Exon V6 Kit (Agilent Technologies, CA, USA) and sequenced on the Illumina HiSeq 4000 platform (Illumina Inc., San Diego, CA, USA). After quality control, raw sequencing reads were aligned to the human reference genome (GRCh37/hg19) using the Burrows-Wheeler Aligner (BWA). Variant annotation was conducted using ANNOVAR.

Variants were filtered as follows: (1) only variants with a minor allele frequency (MAF) <0.01 in public databases, including the 1,000 Genomes Project, NHLBI-ESP, and ExAC, as well as an internal Novogene database, were retained; (2) intergenic, untranslated-region, and deep intronic variants without predicted splice relevance were excluded, while canonical splice-site variants were retained; (3) synonymous variants without predicted functional significance were removed; and (4) the remaining variants were prioritized according to predicted functional relevance, consistency with the suspected inheritance pattern, and a curated gene list related to primary ciliary dyskinesia (PCD). *In silico* tools, including SIFT, PolyPhen-2, MutationTaster, MutationAssessor, and CADD, were used to support variant prioritization. Variant interpretation was performed according to ACMG/AMP guidelines in combination with the patient's phenotype, family segregation, and subsequent RNA and protein validation (11).

### Sanger sequencing

Sanger sequencing was performed to validate the candidate variants identified through whole-exome sequencing. Primer sequences were designed using the online tool provided by Integrated DNA Technologies (IDT) (https://sg.idtdna.com/pages). The specific primer sequences used for amplification and sequencing were as follows: F: CATCATCAACCTCTTGGAGACAG, R: AGCCTCAGTAGTGGACACAT. We amplified the target regions by PCR and sequenced the PCR products using the ABI PRISM 3730 DNA Analyzer (Applied Biosystems) with the BigDye Terminator v3.1 Cycle Sequencing Kit (Applied Biosystems).

### Transmission electron microscopy (TEM)

To investigate ultrastructural abnormalities of respiratory cilia, transmission electron microscopy was performed. Nasal mucosa samples from the proband and a healthy control were fixed in 2.5% glutaraldehyde prepared in 0.1M sodium cacodylate buffer at 4 °C, followed by overnight washing and post-fixation in 1% osmium tetroxide. After a standard dehydration procedure, the samples were embedded in epoxy resin. Ultrathin sections were prepared, mounted on copper grids, and stained with 1% aqueous uranyl acetate and Reynold's lead citrate. Imaging was conducted using a Hitachi AHT7700 transmission electron microscope (Hitachi, Tokyo, Japan) equipped with a MegaView III digital camera (Olympus Soft Imaging Solutions GmbH, Münster, Germany). Ultrastructural evaluation was performed with reference to published consensus criteria for TEM reporting in primary ciliary dyskinesia. More than 50 evaluable axonemal cross-sections with intact ciliary membranes were reviewed (12).

### High-speed video microscopy (HSVM)

Nasal brush biopsy samples from both the proband and the control were suspended in Gibco Medium 199 (12350039, Gibco). Ciliated epithelial strips were visualized using an upright Olympus BX53 microscope (Olympus, Tokyo, Japan) equipped with a 40 × objective lens. Video recordings were captured at room temperature using a scientific CMOS camera (Prime BSI, Teledyne Photometrics Inc., USA) at a frame rate of 50 frames per second (fps). Only intact ciliated edges longer than 50 μm were included for functional analysis.

### Air-liquid interface (ALI) culture

Human nasal respiratory epithelial cells (NECs) were collected from the proband and healthy control using disposable cytology brushes. The cells were seeded onto ALI culture inserts (Corning, USA) and expanded in PneumaCult-Ex Medium (Stemcell Technologies, Canada), followed by differentiation in PneumaCult-ALI Medium (Stemcell Technologies, Canada). Penicillin-streptomycin (Gibco, USA) was added during both the expansion and differentiation phases to prevent microbial contamination.

### Immunofluorescence (IF) analysis

NECs were fixed with 4% paraformaldehyde and permeabilized using 0.1% Triton X-100 to enhance membrane permeability. Non-specific binding sites were blocked with 5% bovine serum albumin (BSA), followed by overnight incubation with primary antibodies at 4 °C. After washing, samples were incubated with fluorophore-conjugated secondary antibodies for 1 hour at room temperature. Nuclei were subsequently counterstained with DAPI, and samples were mounted using an antifade mounting medium. Fluorescence images were acquired and analyzed using a fluorescence microscope (Olympus, Tokyo, Japan). For each marker, more than 10 ciliated cells were examined, and axonemal staining was interpreted qualitatively as present or absent. Detailed information on the primary and secondary antibodies is provided in Supplementary Table 1.

### Real-time quantitative polymerase chain reaction (RT-qPCR)

Total RNA was extracted from NECs of patients and healthy controls using an RNA extraction kit (ER501–01-V2, Full Gold, China). RNA concentration and purity were measured with a NanoDrop 2000 spectrophotometer (Thermo Fisher Scientific, USA). One microgram of total RNA was reverse-transcribed into cDNA using a reverse transcription kit (M1632, Thermo Fisher Scientific, USA). Quantitative PCR was performed using SYBR Green Master Mix (AG11718, Accurate Biology, China) on a 7,500 Fast Real-Time PCR System (Applied Biosystems, USA). The cycling conditions were 95 °C for 30 s, followed by 40 cycles of 95 °C for 5 s and 60 °C for 30 s. GAPDH was used as the internal control, and relative gene expression was calculated using the 2^−Δ*ΔCt*^ method. Six independent culture replicates derived from donor-specific primary cells were analyzed for each group. Primer sequences are provided in Supplementary Table 2.

### Western blot (WB)

Protein lysates were prepared using ice-cold RIPA buffer supplemented with protease and phosphatase inhibitors. Protein concentrations were determined with a BCA assay kit. Equal amounts of protein were separated on 10% SDS-PAGE gels and transferred onto PVDF membranes. After blocking with 5% non-fat milk for 1 h, membranes were incubated overnight at 4 °C with primary antibodies against ZMYND10, DRC1, and GAPDH. Following washes with 0.1% TBST, membranes were incubated with HRP-conjugated secondary antibodies for 1 h at room temperature, and signals were detected using an ECL detection kit (BMU102, Abbkine, China).

### Statistical analyses

Statistical analyses were performed using GraphPad Prism (version 9.0.0). Because this was a single-case study, most experimental findings were interpreted descriptively. For RT-qPCR, six independent culture replicates were used to assess experimental consistency, and the data are presented as individual data points with summary statistics. Categorical variables are presented as counts and percentages.

## Results

### Clinical features

**Figure 1:**
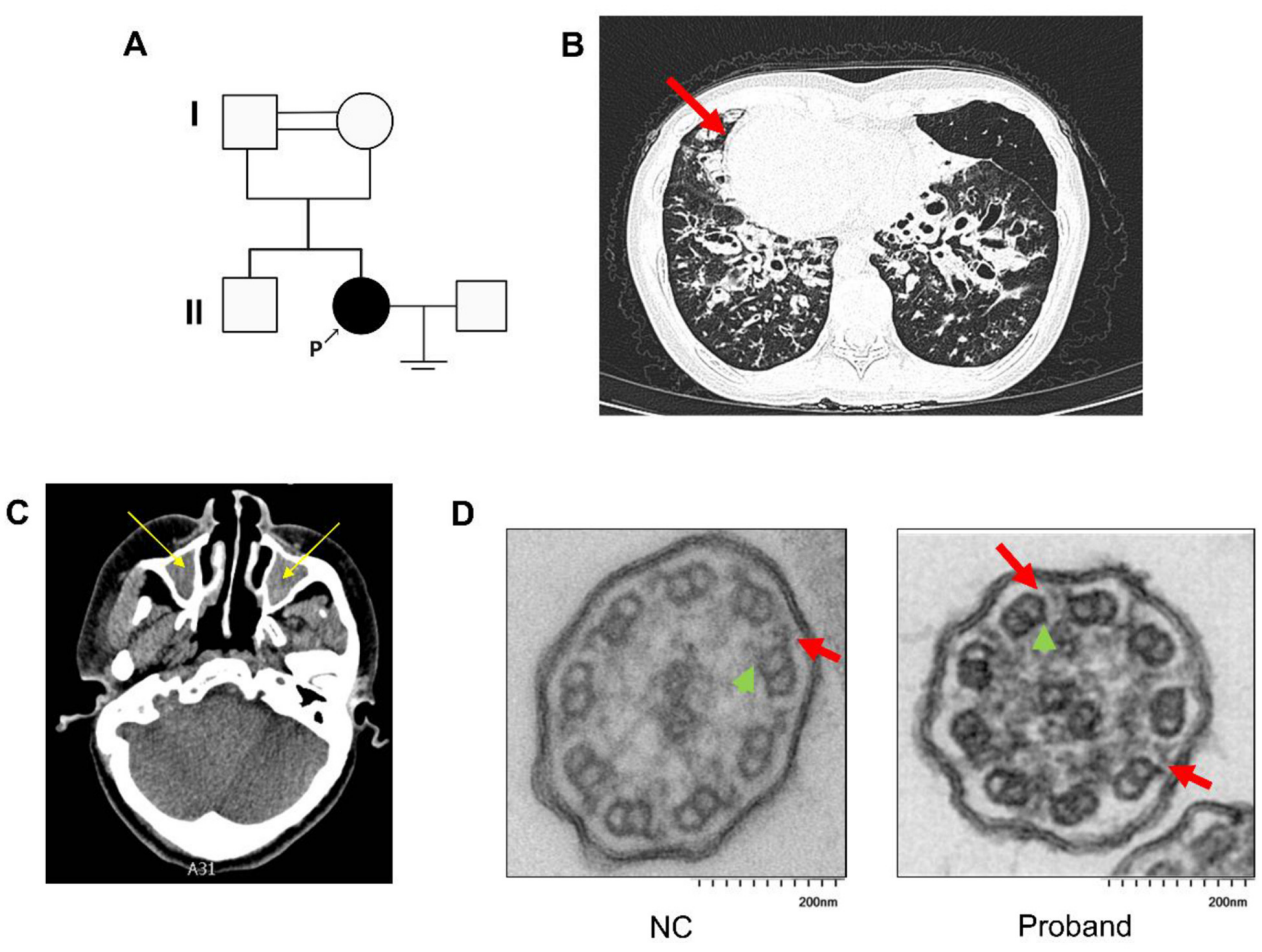
Clinical characteristics and ciliary ultrastructural abnormalities in the proband. **(A)** Pedigree of the proband's family. The filled symbol denotes the proband (P), who is affected by PCD and has a history of primary infertility. **(B)** High-resolution computed tomography (HRCT) of the chest showing bilateral bronchiectasis; the red arrow indicates situs inversus. **(C)** HRCT of the paranasal sinuses demonstrating chronic rhinosinusitis, with mucosal thickening indicated by yellow arrows. **(D)** TEM of respiratory cilia. Compared with the normal control (NC), the proband exhibits loss of both outer dynein arms (red arrows) and inner dynein arms (green arrows). Scale bar, 200 nm.

A 34-year-old woman presented with a history of persistent cough and expectoration of yellow-green sputum for more than 9 years. She also reported anosmia and primary infertility despite 5 years of regular unprotected sexual intercourse. Her parents and brother were asymptomatic and had no related abnormalities (Figure 1A). Chest CT revealed bilateral bronchiectasis (Figure 1B), and sputum culture was positive for *Pseudomonas aeruginosa*. Nasal nitric oxide was markedly reduced to 6.6 nl/min. Paranasal sinus CT showed bilateral chronic sinusitis (Figure 1C). Echocardiography revealed situs inversus with mild mitral and tricuspid regurgitation. Chromosomal analysis showed a normal female karyotype (46, XX).

HSVM of nasal cilia showed near-complete absence of ciliary motion (Supplementary Video 1) compared with the healthy control (Supplementary Video 2). TEM analysis of respiratory cilia showed combined absence or shortening of both ODAs and IDAs in approximately 99% of evaluable axonemal cross-sections (Figure 1D). Taken together, these clinical, radiological, and ultrastructural findings supported a diagnosis of PCD with classic Kartagener syndrome, in keeping with current diagnostic guidelines (13).

### Identification of a *ZMYND10* variant in the proband

**Figure 2:**
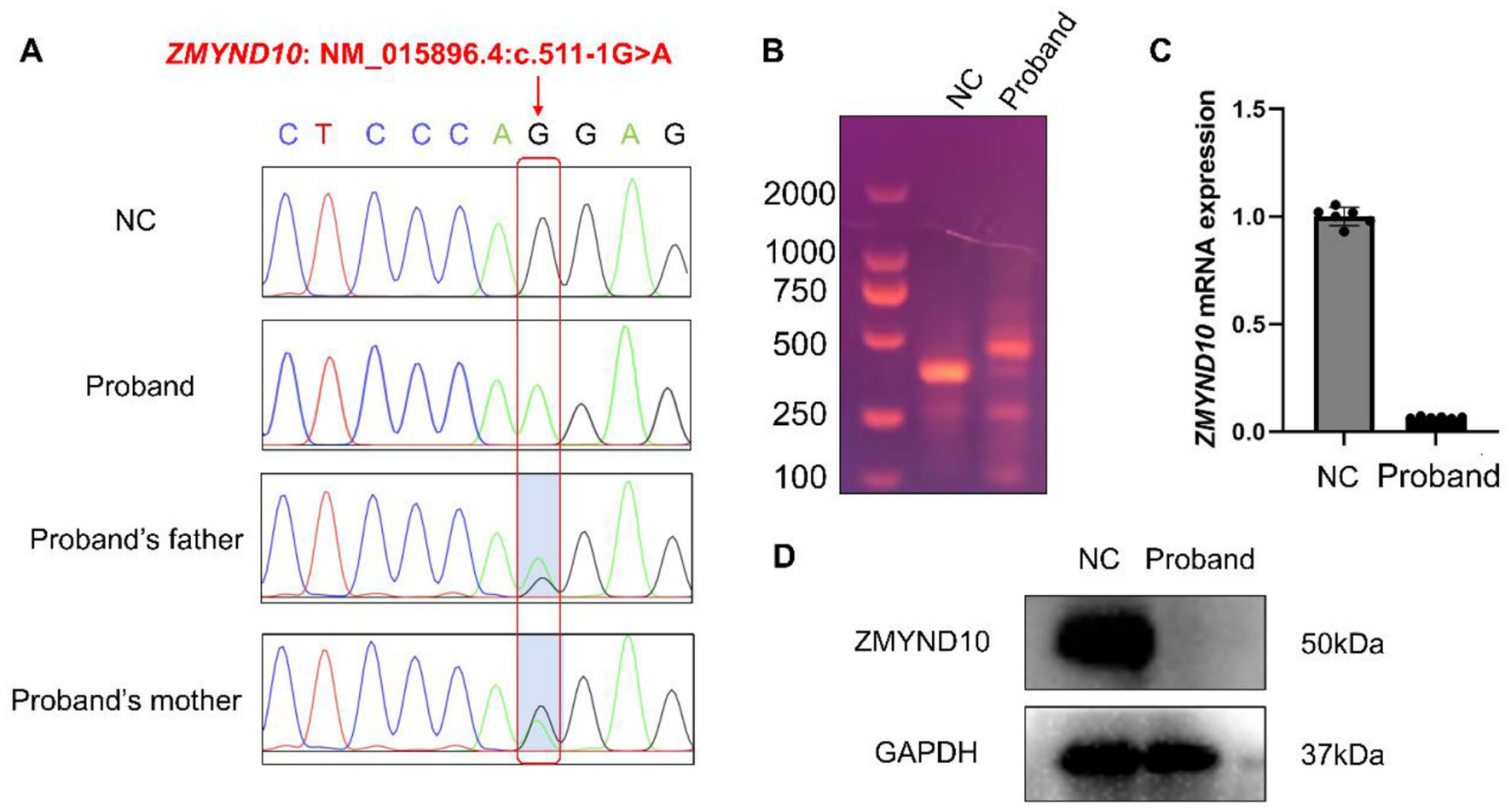
Identification and molecular consequences of a splice-site variant in *ZMYND10*. **(A)** Sanger sequencing chromatograms showing the *ZMYND10* splice-site variant NM_015896.4:c.511–1G>A in the proband; both parents are heterozygous carriers, whereas the normal control (NC) is wild type. **(B)** RT–PCR analysis of *ZMYND10* cDNA demonstrates an aberrant amplicon in the proband compared with the NC, consistent with insertion of an additional 100-bp fragment caused by abnormal splicing. **(C)** RT–qPCR showing markedly reduced *ZMYND10* mRNA levels in the proband, consistent with transcript depletion (e.g., non-sense-mediated mRNA decay). Each dot represents one independent culture replicate derived from donor-specific primary cells. Data are shown as mean ± SD. **(D)** Western blot analysis showing loss of ZMYND10 (~50 kDa) protein expression in the proband; GAPDH (~37 kDa) serves as a loading control. Molecular weight markers (kDa) are shown.

Whole-exome sequencing of peripheral blood DNA identified a homozygous canonical splice-acceptor variant in *ZMYND10* (NM_015896.4:c.511–1G>A). Sanger sequencing confirmed the variant in the proband and demonstrated that both phenotypically unaffected parents were heterozygous carriers (Figure 2A). According to ACMG/AMP guidelines (11), this canonical-1 splice-site variant was interpreted as pathogenic. To assess its effect on mRNA splicing, we first predicted altered splicing patterns using the Rare Disease Data Center (RDDC) platform (Supplementary Figure 1). Subsequently, RNA extracted from the proband's nasal respiratory epithelial cells was reverse-transcribed and analyzed by PCR amplification and Sanger sequencing. Compared with the healthy control, RT-PCR revealed an aberrant cDNA fragment in the proband, and Sanger sequencing confirmed retention of an additional 100-bp intronic sequence (Figure 2B; Supplementary Figure 2). This abnormal splicing event is predicted to cause a frameshift and generate the protein-level consequence NP_056980.2:p.Glu171Valfs^*^3. The insertion introduces a pre-mature termination codon (UAG), which is expected to trigger non-sense-mediated mRNA decay and, if translated, produce a truncated protein lacking the critical C-terminal MYND domain.

### Validation of molecular and structural defects associated with the *ZMYND10* variant

Nasal respiratory epithelial cells (NECs) were collected from the proband and a healthy control using disposable cytology brushes. After 28 days of ALI culture, well-differentiated epithelial cells were obtained. RT-qPCR showed a marked reduction in *ZMYND10* mRNA expression in the proband-derived cells compared with control cells. Western blotting demonstrated complete loss of ZMYND10 protein expression in the proband (Figures 2C, D).

**Figure 3:**
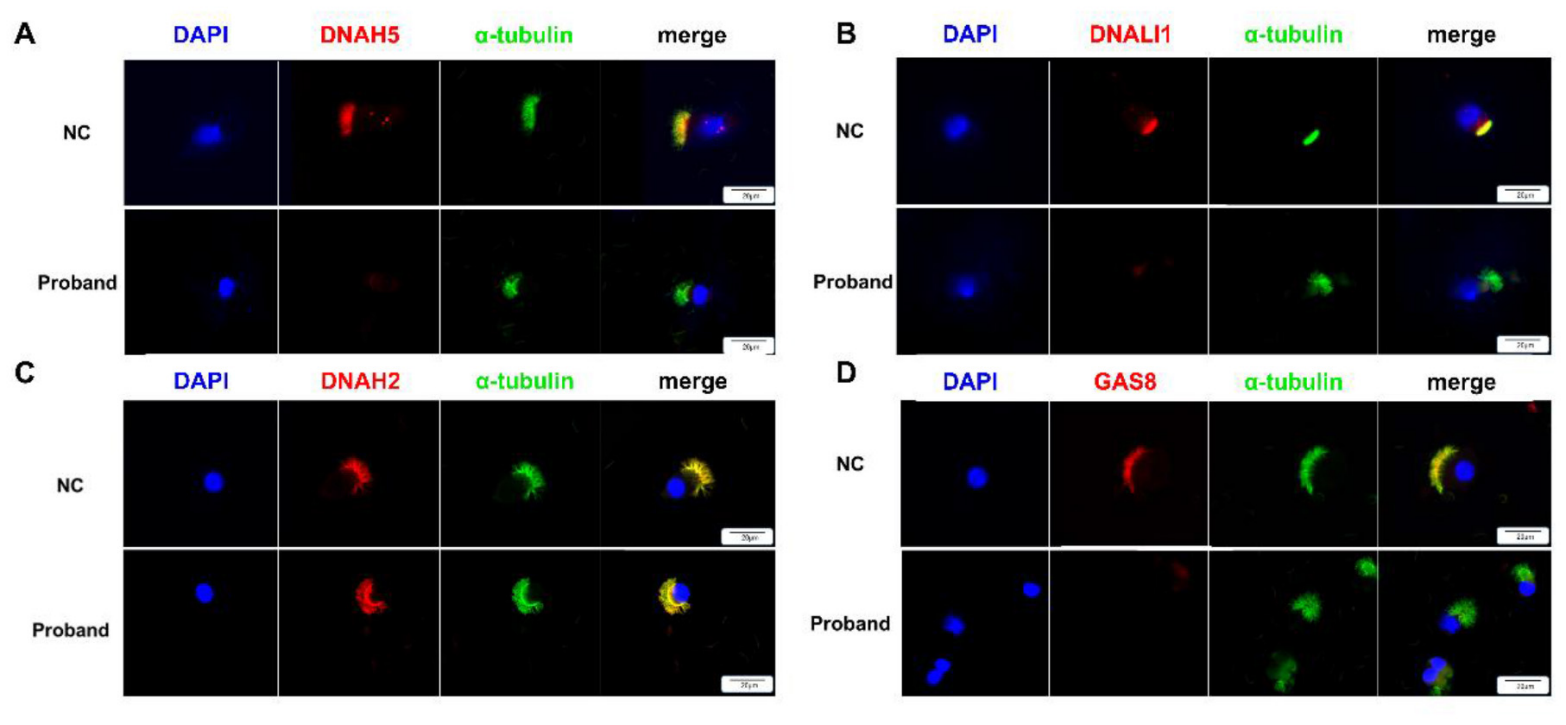
Selective disruption of ODA, single-headed IDA, and GAS8 localization in ZMYND10-deficient airway epithelial cells. **(A)** IF staining of DNAH5 (outer dynein arm marker) in airway epithelial cells. DNAH5 localization along ciliary axonemes is preserved in the normal control (NC) but markedly reduced in the proband. **(B)** IF staining of DNALI1, a single-headed inner dynein arm (IDAa–e) marker, showing clear axonemal localization in the NC but pronounced loss in the proband. **(C)** IF staining of DNAH2, a double-headed inner dynein arm (IDAf) marker, demonstrating comparable axonemal localization in both the NC and the proband. **(D)** IF staining of GAS8 (DRC4) revealing normal axonemal localization in the NC and marked absence in the proband. In all panels, nuclei are stained with DAPI (blue), ciliary axonemes are labeled with α-tubulin (green), and target proteins are shown in red. Merged images are shown in the rightmost column. For each marker, more than 10 ciliated cells were examined, and the images shown are representative of the observed staining pattern. Scale bars, 20 μm.

To further define the impact of the *ZMYND10* variant on ciliary composition, IF staining was performed for selected cilia-associated proteins. For each marker, more than 10 ciliated cells were examined, and a consistent qualitative staining pattern was observed. Compared with the healthy control, the proband exhibited marked loss of the ODA marker DNAH5 (Figure 3A), the single-headed IDA marker DNALI1 (Figure 3B), and the N-DRC marker GAS8 (Figure 3D). Previous studies have shown that loss of DRC1 can disrupt the basal scaffold of the N-DRC and lead to secondary loss of DRC4/GAS8 (14). We therefore examined DRC1 and found that its expression was preserved in the proband (Supplementary Figure 3), suggesting that GAS8 loss cannot be fully explained by DRC1 deficiency alone. In contrast, the IDAf marker DNAH2 remained detectable (Figure 3C). The central pair marker SPEF2 and the radial spoke head marker RSPH3 were also preserved, indicating that these axonemal components remained (Supplementary Figures 5, 6).

Together, these findings indicate an abnormal pattern of axonemal protein localization associated with ZMYND10 deficiency, with loss of some dynein arm- and N-DRC-related markers and preservation of others.

## Discussion

In this study, we present a more comprehensive clinical and molecular characterization of the *ZMYND10* splice-site variant (NM_015896.4:c.511–1G>A) in a patient with classical manifestations of PCD, including chronic rhinosinusitis, bronchiectasis, and situs inversus. Although this case was previously presented by our group in preliminary abstract form (9), the current full-length report substantially extends the available evidence by defining the aberrant splicing consequence at the RNA level and by providing broader protein-level and axonemal characterization. This case also provides clinically supported evidence that primary infertility may be part of the phenotypic spectrum of *ZMYND10*-related PCD, a feature that has only rarely been mentioned in previous reports. In addition, WB and IF demonstrated loss of ZMYND10 together with absence of GAS8 in airway epithelial samples, suggesting that altered N-DRC-associated protein localization may be present (15).

*ZMYND10* was identified in 2013 as a disease gene for PCD and functions as an important factor in dynein arm assembly (7, 8). It is pre-dominantly localized in the cytoplasm and cooperates with LRRC6 to regulate assembly of both outer and inner dynein arms. Loss-of-function variants in *ZMYND10* have been associated with absence of outer dynein arms (ODAs) and inner dynein arms (IDAs) in airway epithelial cilia, consistent with our findings. Nevertheless, the precise effects of *ZMYND10* deficiency on specific axonemal substructures remain incompletely defined.

Human respiratory IDAs comprise six single-headed structures (IDA a–e and g) and one double-headed structure (IDAf)(16, 17). In our study, ZMYND10 deficiency was associated with loss of DNALI1, a component of the single-headed IDAs, whereas DNAH2, a component of double-headed IDAf, was preserved. This pattern suggests that ZMYND10 may exert a greater impact on single-headed IDA assembly than on double-headed IDAf. Unfortunately, because of limited antibody availability, we were unable to evaluate CETN2 or DNAH6, which are related to IDAg, and the precise boundaries of this selective effect remain to be clarified. Mechanistic studies of ZMYND10 have so far focused mainly on ODA assembly. ZMYND10 participates in this process through interaction with LRRC6 (7) and through the ZMYND10–FKBP8–HSP90 chaperone relay (18). These pathways may be differentially affected depending on whether a specific variant disrupts the MYND domain, which is critical for protein–protein interactions. In *Zmynd10* knockout mice, *Dnai1* and *Dnali1* mRNA levels are not reduced relative to wild-type controls, and *Zmynd10* physically interacts with *Dnai1* (19). Collectively, the mouse model data and human protein interaction studies indicate that *ZMYND10* primarily supports dynein arm pre-assembly or stability at the protein level rather than directly regulating transcription. Consistent with this model, destabilization of the ZMYND10-associated chaperone network can lead to stalled dynein pre-assembly or degradation of incompletely assembled subunits, ultimately resulting in ODA loss (20). Taken together with previous studies, our findings support a role for ZMYND10 in dynein arm pre-assembly or stability at the protein level. The structural abnormalities observed here are consistent with disturbed dynein assembly homeostasis.

Notably, phenotypic variability associated with *Zmynd10* deficiency has also been observed in murine models. In two independent studies using distinct *Zmynd10* knockout strategies, divergent ciliary phenotypes were reported: one model showed a normal number of ependymal cilia that were largely immotile (18), whereas the other showed a marked reduction in ciliary number (21). These discrepancies may reflect differences in the targeted genomic regions and the residual protein domains retained, supporting a potential domain- or allele-specific role of *ZMYND10* in ciliogenesis and ciliary motility.

Such observations may help explain the heterogeneity seen among individuals with *ZMYND10* variants. In particular, primary infertility in affected women may be influenced by variant-specific effects on protein function, although this possibility remains to be clarified. Our findings, together with prior animal data (18, 21), raise the possibility that distinct ZMYND10 variants may be associated with variable effects on ciliary assembly, stability, and motility, thereby contributing to variable respiratory and reproductive phenotypes.

We also observed loss of GAS8 (DRC4) in this patient despite preserved DRC1, indicating that GAS8 loss cannot be fully explained by DRC1 deficiency alone (14). This pattern suggests that ZMYND10 dysfunction may be associated with altered N-DRC organization or axonemal protein stability, particularly given the established role of GAS8 as a core N-DRC component (22). These findings expand the molecular features currently recognized in ZMYND10-related PCD and warrant further investigation.

A plausible explanation for infertility in women with PCD is impaired cilia-mediated transport within the fallopian tubes, which may hinder ovum or embryo transport to the uterine cavity (23, 24). Male infertility is well recognized in PCD and has also been described in association with ZMYND10-related disease (8, 18); however, the reproductive implications of *ZMYND10* variants in women have received far less attention (7, 8, 25) (Supplementary Table 4). The relative scarcity of reported cases may reflect the young age of many patients, the difficulty of directly assessing ciliary function in the female reproductive tract, and incomplete long-term follow-up. These considerations support incorporation of reproductive assessment and fertility counseling into the multidisciplinary care of women with PCD. However, the reproductive implications remain inferential in the absence of direct functional assessment of cilia in the female reproductive tract. In our case, the proband was advised to seek consultation with a fertility specialist and to consider medically assisted reproduction if clinically appropriate (23). However, because of the severity of her respiratory manifestations, she has not yet pursued reproductive intervention.

This study has several limitations. First, the downstream molecular and axonemal analyses were based on a single patient-control comparison, and the observed differences should therefore be interpreted as case-based findings rather than definitive evidence of a generalizable mechanism. Second, only a limited number of axonemal markers were examined, and additional components could not be assessed because of antibody availability. Third, although our observations are biologically consistent with previous studies of ZMYND10, the mechanistic implications of the present findings require validation in additional patients and functional models.

In summary, we provide a detailed clinical and molecular characterization of the *ZMYND10* splice-site variant associated with classical PCD features, primary infertility, and altered localization of dynein arm and N-DRC components. These findings refine genotype–phenotype correlations in *ZMYND10*-related PCD and suggest possible variant-specific effects on ciliary assembly, stability, and motility. More broadly, this case expands the recognized clinical and molecular spectrum of *ZMYND10* deficiency and highlights the importance of reproductive assessment in affected women.

## Data Availability

The original contributions presented in the study are included in the article/Supplementary material, further inquiries can be directed to the corresponding authors.
