# Supplementary material for: A homozygous splice-site variant in ZMYND10 causes primary ciliary dyskinesia with primary infertility in a Chinese family
Source: Front Med (Lausanne). 2026 May 8;13:1838497. doi: 10.3389/fmed.2026.1838497 (PMC13194126; doi:10.3389/fmed.2026.1838497)
Supplement: Supplementary file 1 [file Supplementary_file_1.docx]

**A homozygous splice-site variant in *ZMYND10* causes primary ciliary dyskinesia with female infertility in a Chinese family**

Jie He^1,2,3,4,†^, Xiangyang Lu^1,2,3,4,†^, Manqing Guo^1,2,3,4^, Binyi Yang^1,2,3,4^, Xianglin Zhou^1,2,3,4^, Ying Liu^1,2,3,4^, Hui Fan^1,2,3,4^, Danhui Yang^1,2,3,4,*^, Hong Luo^1,2,3,4,*^

1 Department of Pulmonary and Critical Care Medicine, the Second Xiangya Hospital, Central South University, Changsha, Hunan 410011, China

2 Research Unit of Respiratory Disease, Central South University, Changsha, Hunan 410011, China

3 Clinical Medical Research Center for Pulmonary and Critical Care Medicine in Hunan Province, Changsha, Hunan 410011, China

4 Diagnosis and Treatment Center of Respiratory Disease in Hunan Province, Changsha, Hunan 410011, China

*CORRESPONDENCE

Hong Luo

Email: [luohonghuxi@csu.edu.cn](mailto:luohonghuxi@csu.edu.cn)

Danhui Yang

Email: [188202086@csu.edu.cn](mailto:188202086@csu.edu.cn)

^†^These authors have contributed equally to this work.


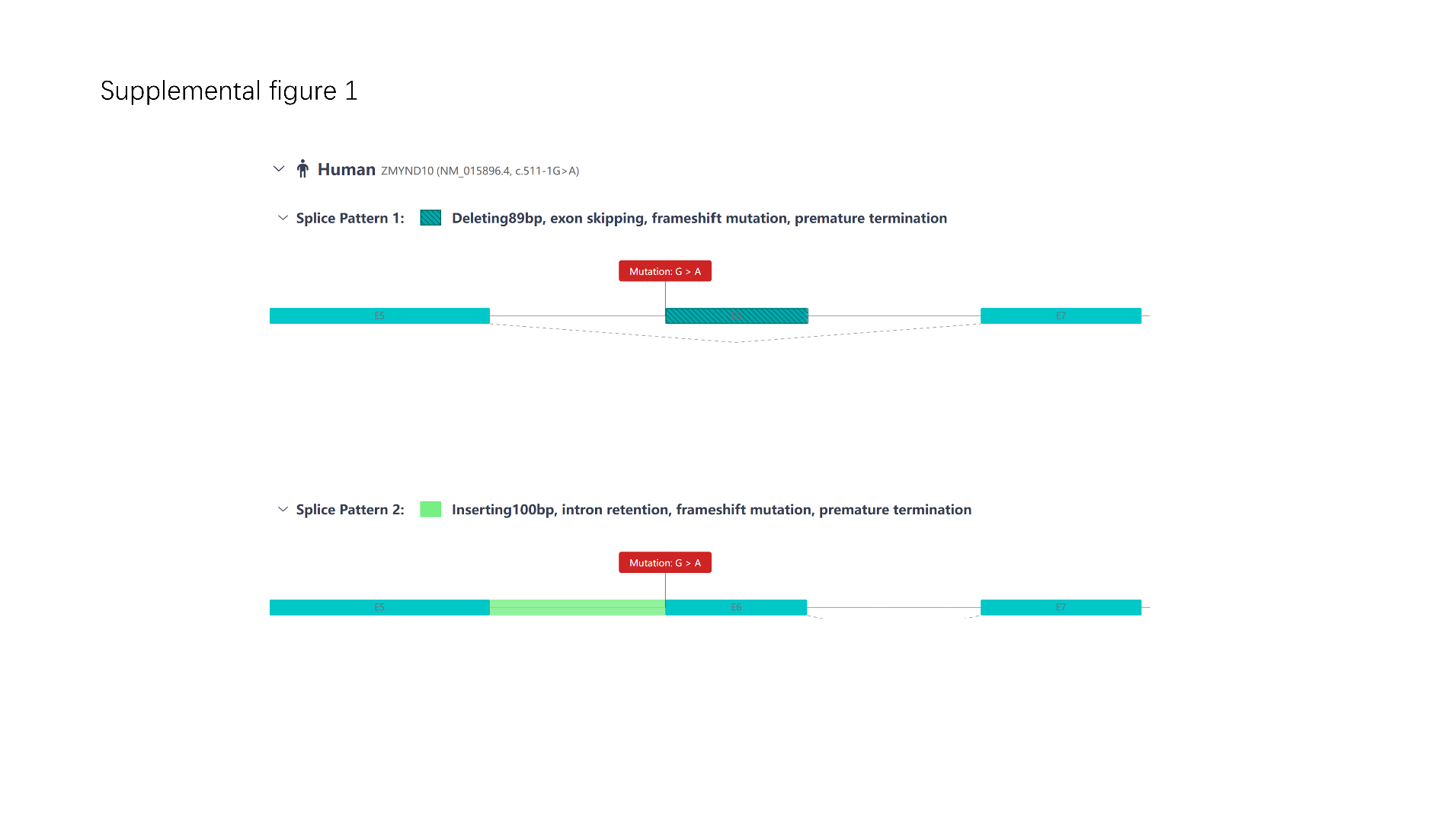


**Supplementary Figure 1. In silico prediction of aberrant splicing caused by the *ZMYND10* variant.**

RDDC-based splicing prediction for *ZMYND10* (NM_015896.4:c.511−1G>A) indicating two potential abnormal splicing outcomes: (i) exon skipping with an 89-bp deletion, and (ii) intron retention with a 100-bp insertion, both predicted to result in a frameshift and premature termination.


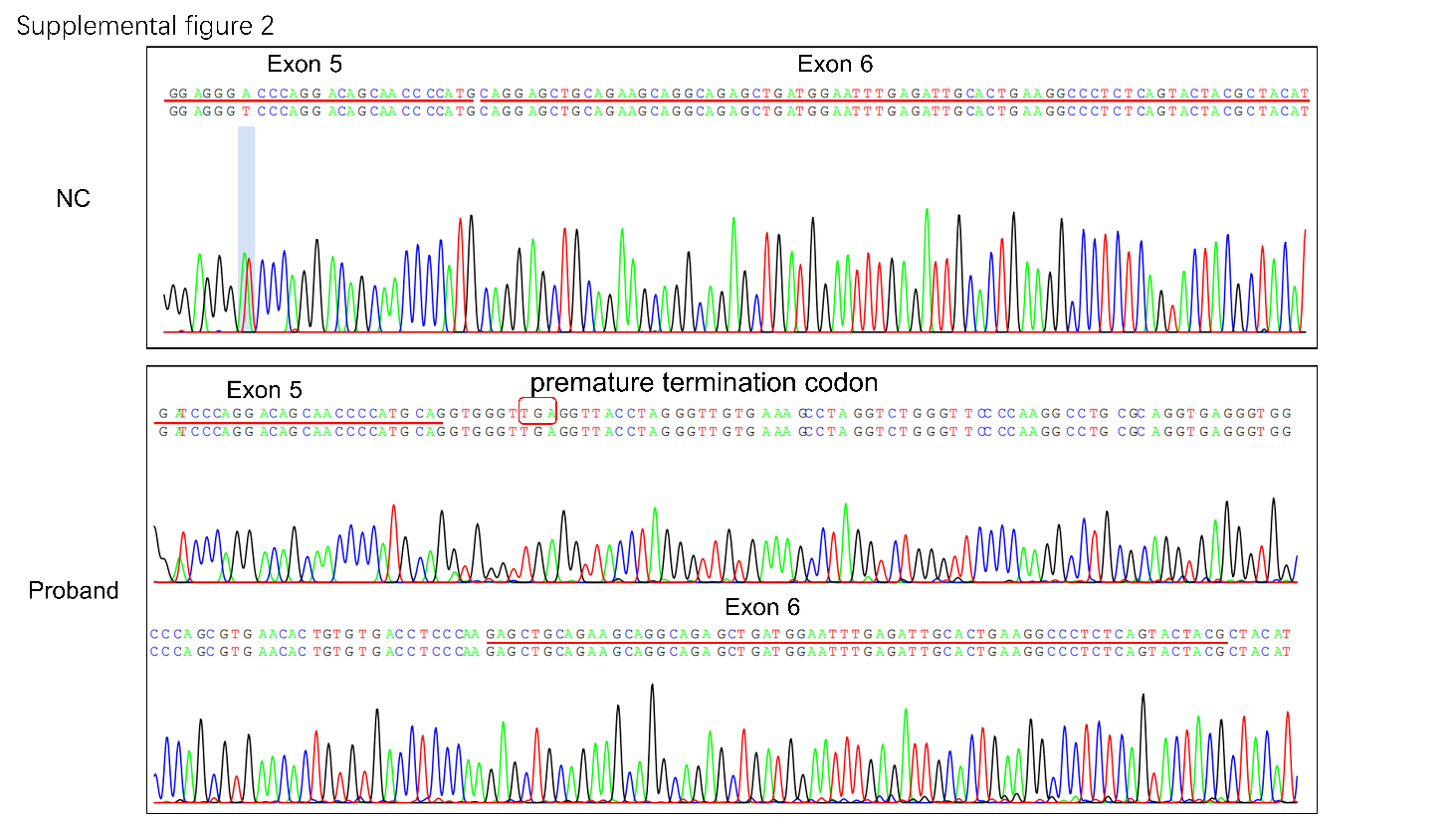


**Supplementary Figure 2. cDNA sequencing confirms aberrant *ZMYND10* splicing and premature termination.**

Sanger sequencing chromatograms of *ZMYND10* cDNA showing a normal exon 5–exon 6 junction in the normal control (NC), whereas the proband exhibits aberrant splicing with generation of a premature termination codon.


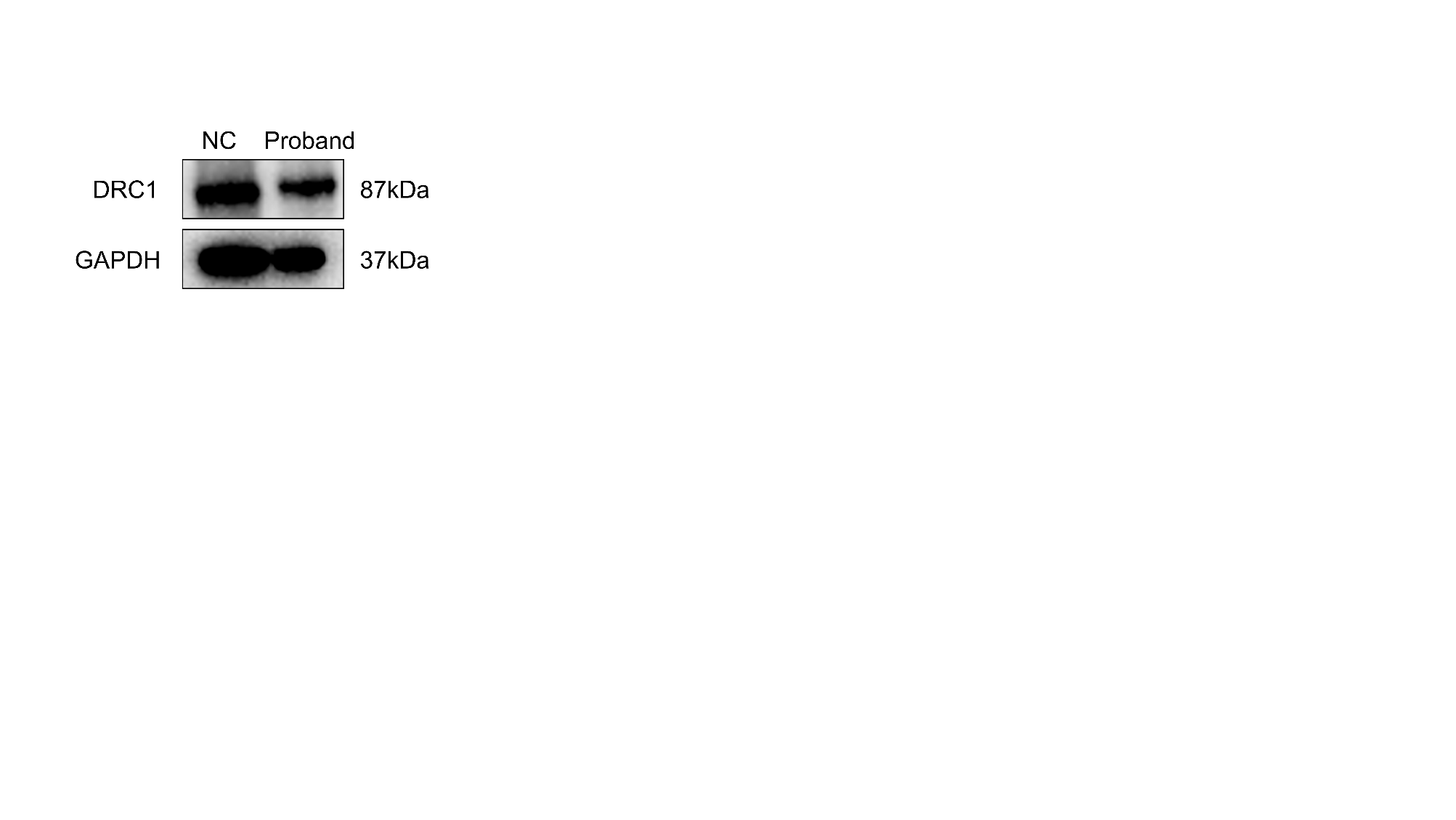


**Supplementary Figure 3. DRC1 protein expression is preserved in the proband.**

Western blot analysis showing DRC1 (~87kDa) expression in the proband comparable to the NC. GAPDH (~37kDa) serves as a loading control. Molecular weight markers (kDa) are shown.


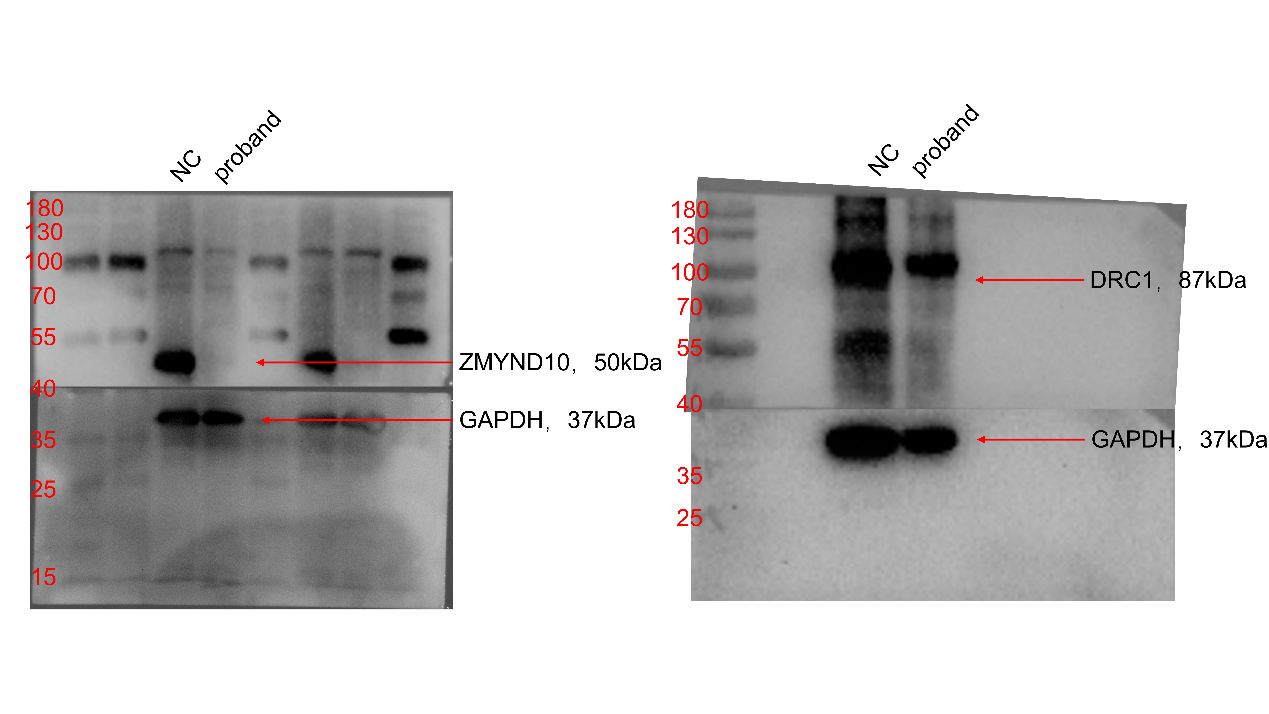


**Supplementary Figure 4. Uncropped western blot images.**
Full-length western blot images showing ZMYND10 (~50 kDa) and DRC1 (~87 kDa) expression in the normal control (NC) and the proband, with GAPDH (~37 kDa) as a loading control. Molecular weight markers (kDa) are shown.


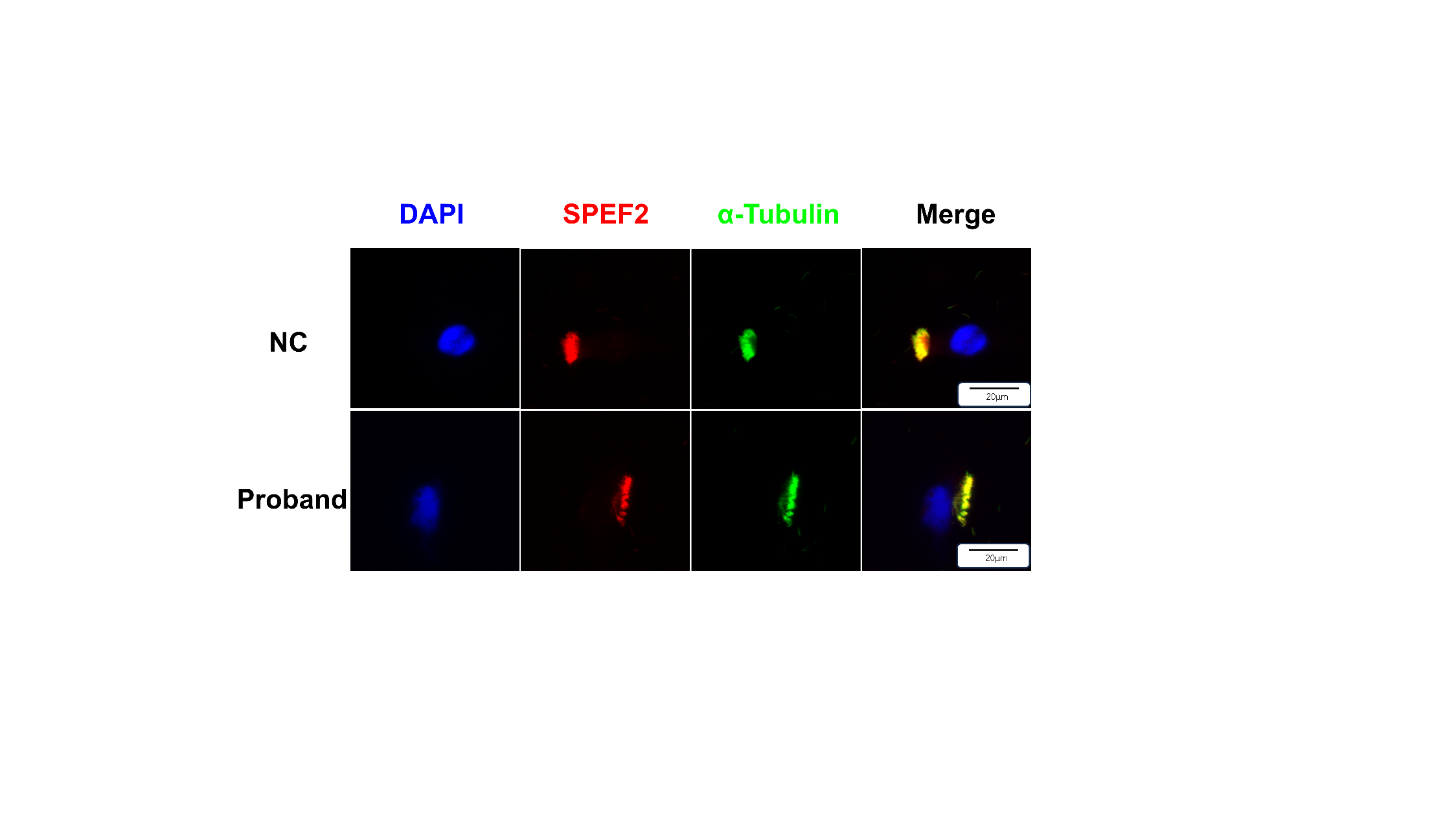


**Supplementary Figure 5. Axonemal localization of SPEF2 is maintained in the proband.**

IF staining of SPEF2 in airway epithelial cells from the NC and the proband. Nuclei are stained with DAPI (blue), ciliary axonemes are labeled with α-tubulin (green), and SPEF2 is shown in red. Merged images are shown. Scale bar, 20 μm.


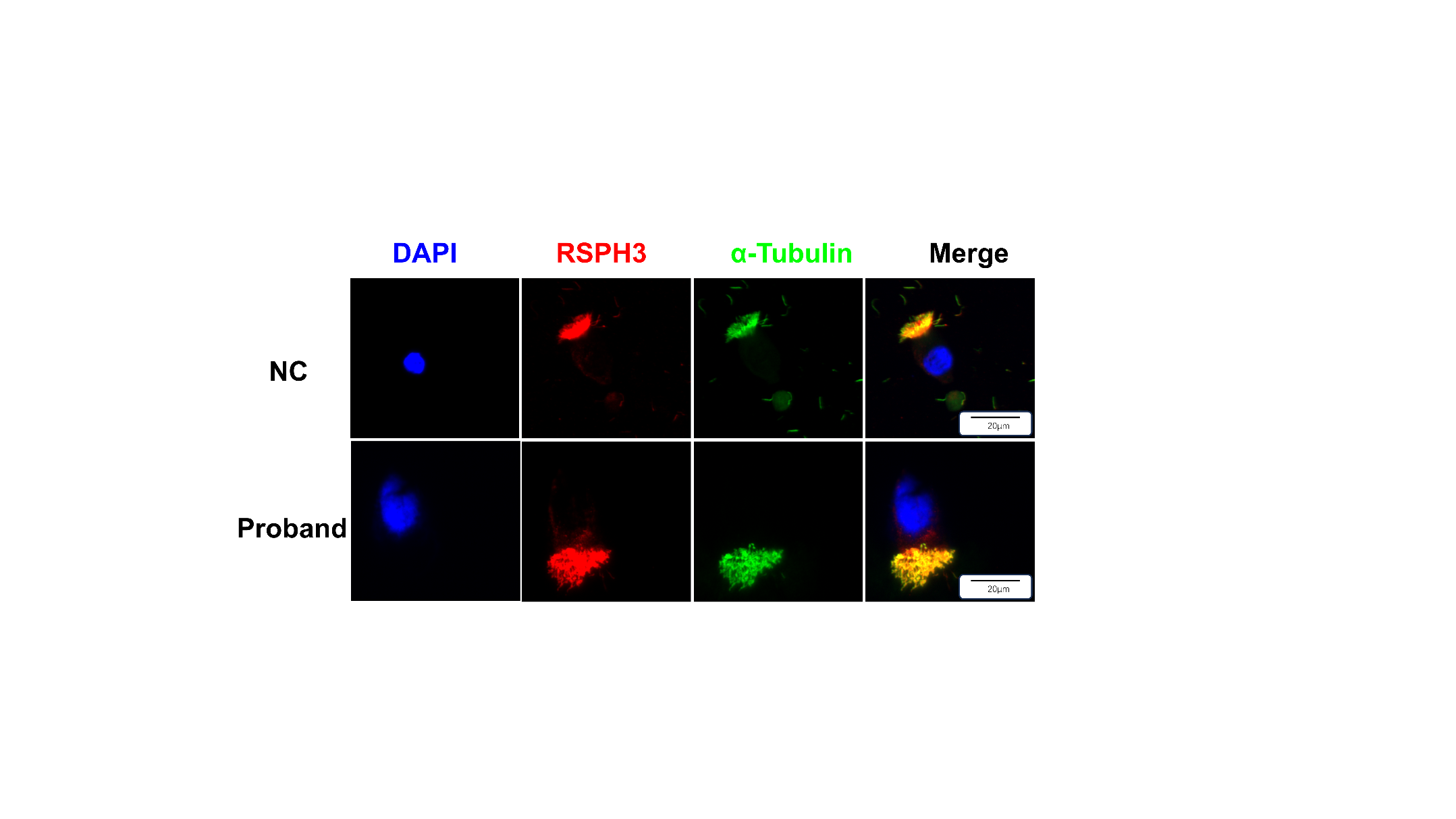


**Supplementary Figure 6. Axonemal localization of RSPH3 is maintained in the proband.**

IF staining of RSPH3 in airway epithelial cells from the NC and the proband. Nuclei are stained with DAPI (blue), ciliary axonemes are labeled with α-tubulin (green), and RSPH3 is shown in red. Merged images are shown. Scale bar, 20 μm.

**Supplemental table 1. Antibodies used in this study.**

| Target | Host/Clone | Vendor | Catalog No. | Application | Dilution |
| --- | --- | --- | --- | --- | --- |
| α-tubulin | Mouse/monoclonal | Sigma | T9026 | IF | 1:400 |
| DNAH5 | Rabbit/Polyclonal | Sigma | HPA037470 | IF | 1:50 |
| DNAH2 | Rabbit/Polyclonal | Sigma | HPA067103 | IF | 1:200 |
| DNALI1 | Rabbit/Polyclonal | Thermo Fisher Scientific | PA5-97673 | IF | 1:25 |
| GAS8 | Rabbit/Polyclonal | Sigma | HPA041311 | IF | 1:100 |
| SPEF2 | Rabbit/Polyclonal | Sigma | HPA039606 | IF | 1:50 |
| RSPH3 | Rabbit/Polyclonal | Proteintech | 17603-1-AP | IF | 1:100 |
| GAPDH | Rabbit/Polyclonal | Proteintech | 10494-1-AP | WB | 1:10000 |
| ZMYND10 | Rabbit/Polyclonal | Proteintech | 14431-1-AP | WB | 1:1000 |
| DRC1 | Rabbit/Polyclonal | Proteintech | 26219-1-AP | WB | 1:1000 |

Note: Secondary antibodies: IF—Alexa Fluor 488 goat anti-mouse IgG (Thermo Fisher Scientific, A21121; 1:1,000) and Alexa Fluor 555 goat anti-rabbit IgG (Thermo Fisher Scientific, A32732; 1:1,000); WB—HRP-conjugated goat anti-rabbit IgG (Proteintech, SA00001-2; 1:10,000).

**Supplemental table 2. Primer sequences for splicing verification**

| Target | Primer sequence (5’→3’) | Product size (bp) |
| --- | --- | --- |
| *ZMYND10* | *ZMYND10*-splicing-F | CACCGCAAACTGACCCTGCT |
| *ZMYND10* | *ZMYND10*-splicing-R | CGATCCACACTTGCCCGTCCA |

**Supplemental table 3. Primer sequences for RT-qPCR.**

| Target | Primer sequence (5’→3’) | Product size (bp) |
| --- | --- | --- |
| *GAPDH* | *GAPDH*-rtpcr-F | CAGGAGGCATTGCTGATGAT |
| *GAPDH* | *GAPDH*-rtpcr-R | GAAGGCTGGGGCTCATTT |
| *ZMYND10* | *ZMYND10*-rtpcr-F | GACAGTGTTCTTCCACAAGGAGGT |
| *ZMYND10* | *ZMYND10*-rtpcr-R | CAGTTTGCGGTGGCAATAGTCT |

**Supplemental table 4. A chronological summary of published cases on *ZMYND10* mutant patients**

| Reference | Sex | Variants | Age at diagnosis | nNO | Bronchiectasis/bronchitis | Sinusitis | Laterality defects | fertility |
| --- | --- | --- | --- | --- | --- | --- | --- | --- |
| Zariwala et al. | M | c.47T>G  (p.Val16Gly) | NA | NA | Y | Y | Y | N |
|  | F | c.47T>G  (p.Val16Gly) c.300delC  (p.Phe101Serfs^∗^38) | NA | NA | Y | Y | N | N |
|  | F | c.85T>C  (p.Ser29Pro) | NA | NA | Y | Y | Y | N |
|  | M | c.85T>C  (p.Ser29Pro) | NA | NA | Y | Y | Y | Y |
|  | M | c.300delC  (p.Phe101Serfs^∗^38) | NA | NA | Y | Y | N | N |
|  | F | c.486dupA  (p.Ser163Ilefs^∗^20) | NA | NA | Y | Y | N | N |
|  | M | c.dup608_609dupTC  (p.Thr205Alafs^∗^3) | NA | NA | Y | Y | Y | N |
|  | F | c.dup608_609dupTC  (p.Thr205Alafs^∗^3) | NA | NA | Y | Y | Y | N |
|  | M | c.683G>A  (p.Trp228^∗^) | NA | NA | Y | Y | NA | N |
|  | M | c.683G>A  (p.Trp228^∗^) | NA | NA | Y | Y | NA | N |
|  | M | NA (homozygous deletion, exons 7-12) | NA | NA | Y | Y | N | N |
|  | M | c.967C>T  (p.Gln323^∗^) | NA | NA | Y | Y | Y | N |
|  | M | c.967C>T  (p.Gln323^∗^) | NA | NA | Y | Y | Y | N |
|  | M | c.1038_1039delAG  (p.Gly347Glnfs^∗^30) | NA | NA | Y | Y | Y | N |
|  | F | c.1096C>T  (p.Gln366^∗^) | NA | NA | Y | Y | N | N |
|  | M | c.1136A>G  (p.Tyr379Cys) | NA | NA | N | N | Y | N |
| Moore et al. | F | c.47T>G  (p.Val16Gly) | 8y | 23ppb^a^ | Y | Y | N | Y |
|  | F | c.47T>G  (p.Val16Gly)  c.589_590delTG  (p.Val198Glyfs*13) | 2y | NA | Y | Y | Y | NA |
|  | F | c.797T>C  (p.Leu266Pro) | NA | NA | NA | Y | Y | NA (had IVF) |
|  | F | c.797T>C  (p.Leu266Pro) | NA | NA | NA | NA | N | NA |
|  | M | NA | NA | NA | NA | NA |  | NA |
|  | F | c.47T>G  (p.Val16Gly) | NA | NA | NA | NA | N | NA |
|  | F | c.47T>G  (p.Val16Gly) | NA | NA | NA | NA | N | NA |
|  | F | c.65delT  (p.Phe22Serfs*21) | 4mo | NA | NA | Y | Y | NA |
|  | F | NA | 3mo | NA | NA | Y | Y | NA |
|  | M | c.47T>G  (p.Val16Gly) c.116T>C  (p.Leu39Pro) | 3y | 32ppb^a^ | N | Y | Y | NA |
| Kurkowiak et al. | M | c.367delC  (p.H123Tfs*16) | NA | 23ppb^b^ | Y | Y | N | NA |
|  | F | c.367delC  (p.H123Tfs*16) | NA | 117ppb^b^ | Y | Y | Y | NA |
| Ozkavukcu et al. | M | c.386delC  (p.Ser129*) | NA | NA | Y | Y | Y | Y |

a. The nNO value was reported without a corresponding normal reference range, and the sampling flow rate was not specified. b. The normal reference range for the nNO value is 450–760 ppb. Y: YES; N: NO; NA: not applicable. nNO: nasal nitric oxide. IVF: in vitro fertilization.

**Supplementary Video 1. High-speed video microscopy assessment of respiratory ciliary motion in the proband.**High-speed video microscopy was used to evaluate ciliary beating activity in respiratory epithelial cells obtained from the proband. The cilia showed markedly impaired motility and were essentially immotile during observation.

**Supplementary Video 2. High-speed video microscopy assessment of respiratory ciliary motion in a healthy control.**High-speed video microscopy was used to evaluate ciliary beating activity in respiratory epithelial cells obtained from a healthy control. The cilia exhibited regular, coordinated, and rhythmic beating during observation, consistent with normal ciliary motility.
